# Crystal structure and luminescent properties of bis­[2,6-dimethyl-3-(pyridin-2-yl-κ*N*)pyridin-4-yl-κ*C* ^4^](2,2,6,6-tetra­methylhepta­ne-3,5-dionato-κ^2^ *O*,*O*′)iridium(III) ethyl acetate monosolvate

**DOI:** 10.1107/S2056989018011076

**Published:** 2018-08-10

**Authors:** Ki-Min Park, Suk-Hee Moon, Youngjin Kang

**Affiliations:** a Research Institute of Natural Science, Gyeongsang National University, Jinju 52828, Republic of Korea; b Department of Food and Nutrition, Kyungnam College of Information and Technology, Busan 47011, Republic of Korea; c Divisionof Science Education, Kangwon National University, Chuncheon 24341, Republic of Korea

**Keywords:** crystal structure, iridium(III) complex, *C*,*N*-chelating ligand, C—H⋯π inter­actions, luminescence

## Abstract

In the solvated title compound, [Ir(C_12_H_11_N_2_)_2_(C_11_H_19_O_2_)]·CH_3_CO_2_C_2_H_5_, the Ir^III^ ion adopts a distorted octa­hedral coordination environment resulting from its coordination by two *C*,*N*-chelating 2,6-dimethyl-3-(pyridin-2-yl)pyridin-4-yl ligands and one *O*,*O′*-chelating 2,2,6,6-tetra­methylhepta­ne-3,5-dionate ligand. The *C*,*N*-chelating ligands are perpendicular to each other [dihedral angle between the least-squares planes = 87.86 (5)°] and are arranged in a *cis*-*C*,*C′* and *trans*-*N*,*N′* fashion. In the crystal, pairwise C—H⋯π inter­actions between inversion-related Ir^III^ complexes are present, forming a dimeric structure. The title complex shows bright bluish–green emission with good quantum efficiency in solution at room temperature.

## Abstract 2

The Ir^III^ atom in the solvated title complex adopts a distorted octa­hedral geometry coordinated by two *C*,*N*-chelating 2,6-dimethyl-3-(pyridin-2-yl)pyridin-4-yl ligands and one *O*,*O*′-chelating 2,2,6,6-tetra­methylhepta­ne-3,5-dionate ligand. The title compound shows bright blue–green emission in solution at room temperature.

## Chemical context   

Bi­pyridine-based iridium(III) complexes have recently attracted much attention because of their applications in organic light-emitting diodes (OLEDs) (Kim *et al.*, 2018*a* ▸; Reddy *et al.*, 2016 ▸). In particular, fluorinated- or alkoxo-functionalized bi­pyridine ligands have attracted increasing inter­est in materials research fields because of their large energy differences (*T*_1_→*S*_0_) between the triplet (*T*_1_) excited states and singlet ground states (Kim *et al.*, 2017 ▸). This large triplet energy makes them useful and effective ligands for the design of blue phospho­rescent metal complexes. Inter­estingly, Ir^III^ complexes bearing either meth­oxy or isoprop­oxy substituents in *C*-coordinating pyridine show blue emission at room temperature, although these substituents act as electron-donating groups (Lee *et al.*, 2014 ▸; Kim *et al.*, 2018*b* ▸). This could be due to their large triplet energy (*T*_1_ = 2.70–2.82 eV). Compared with alk­oxy substituents, the methyl group has been regarded as essentially the same substituent because of its electron-donating nature. However, an Ir^III^ complex based on methyl-substituted bi­pyridine as a main ligand emits strong green phospho­rescence emission at room temperature (Kim *et al.*, 2017 ▸). This fact prompted us to investigate the structure of a new Ir^III^ compound possessing methyl-substituted bi­pyridine ligands because the emission of the Ir^III^ complex is dependent on both the main ligand and the structural diversity of the metal complex. Herein, we describe the results of our investigation of the crystal structure, thermal and luminescent properties of the title solvated Ir^III^ complex possessing methyl-substituted bi­pyridine, which was motivated by its potential application for OLEDs.

## Structural commentary   

**Figure 1:**
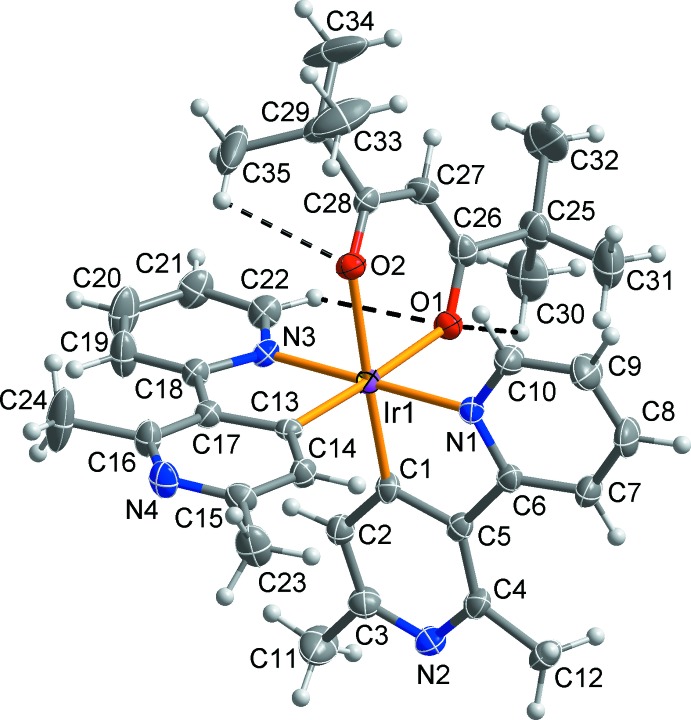
The mol­ecular structure of the title compound, with the atom-numbering scheme. Displacement ellipsoids are drawn at the 50% probability level and H atoms are shown as circles of arbitrary radii. Yellow dashed lines represent intra­molecular C—H⋯O hydrogen bonds. The ethyl acetate solvent mol­ecule is not shown for clarity.

As shown in Fig. 1 ▸, the asymmetric unit of the title compound consists an Ir^III^ cation, two 2,6-dimethyl-3-(pyridin-2-yl)pyridin-4-yl ligands and a 2,2,6,6-tetra­methylhepta­ne-3,5-dionate ligand. The Ir^III^ atom has a distorted octa­hedral coordination sphere defined by two *C*,*N*-chelating ligands and one *O*,*O′*-chelating ligand. The *C*,*N*-chelating ligands, which are almost perpendicular to each other [dihedral angle between the least-squares planes = 87.86 (5)°], are arranged in *cis*-*C*,*C′* and *trans*-*N*,*N′* fashions. These arrangements are similar to those in [Ir(ppy)_2_(acac)] (Adachi *et al.*, 2001 ▸) and [Ir(dfpypy)_2_(acac)] (Kang *et al.*, 2013 ▸) where the ppy, dfpypy and acac ligands are 2-phenyl­pyridinate, 2′,6′-di­fluoro-2,3′-bipyridinate, and acetyl­acetonate, respectively. Within the bi­pyridine ligands, the pyridine rings are approximately co-planar, with the dihedral angles between the N1/C6–C10 and N2/C1–C5 rings being 12.49 (19)° and that between rings N3/C18–C22 and N4/C13–C17 being 4.82 (12)°, indicating that effective π conjugation of the two pyridine rings occurs in the ligands.

The Ir—N, Ir—C and Ir—O bond lengths (Table 1 ▸) are typical for related octa­hedrally coordinated Ir^III^ complexes, for example, bis­[2-*tert*-but­oxy-6-fluoro-3-(pyridin-2-yl-*κN*)pyridin-4-yl-*κC* ^4^](pentane-2,4-dionato-*κ* ^2^ *O*,*O′*)iridium(III) (Park & Kang, 2014 ▸), bis­[2-(1,3-benzo­thia­zol-2-yl)phenyl-*κ* ^2^ *C* ^1^,*N*][1,3-bis­(4-bromo­phen­yl)propane-1,3-dionato-*κ* ^2^ *O*,*O′*]iridium(III) (Kim *et al.*, 2013 ▸) or (acetyl­acetonato-*κ* ^2^ *O*,*O′*)bis[3-(2-pyrid­yl)-2,6-di­fluoro-4-pyridyl-*κ* ^2^ *C*,*N*]iridium(III) (Kang *et al.*, 2013 ▸). The average length [1.976 (3) Å] of the Ir—C bonds is slightly shorter than that [2.030 (2) Å] of the Ir—N bonds because of back bonding between the metal atom and an anionic C atom of the ligand. Weak intra­molecular C—H⋯O inter­actions between the 2,2,6,6-tetra­methylhepta­ne-3,5-dionate O atoms as acceptors and the C22—H22, C30—H30*A* and C35–H35*A* groups as donors (Table 2 ▸, dashed lines in Fig. 1 ▸) contribute to the stabilization of the Ir^III^ complex.
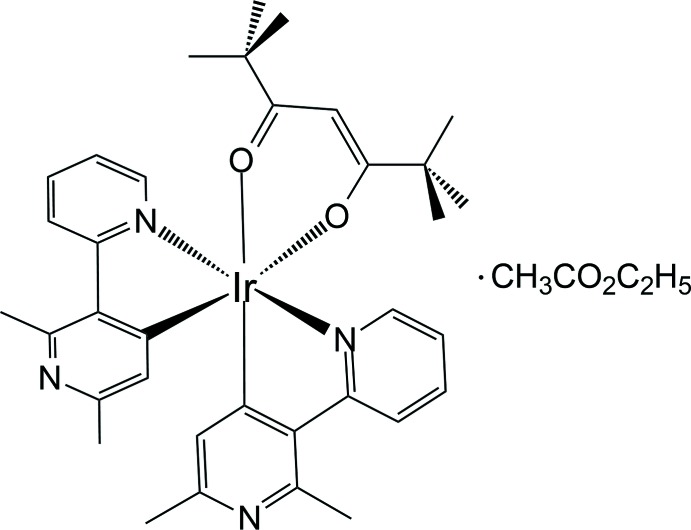


## Supra­molecular features   

**Figure 2:**
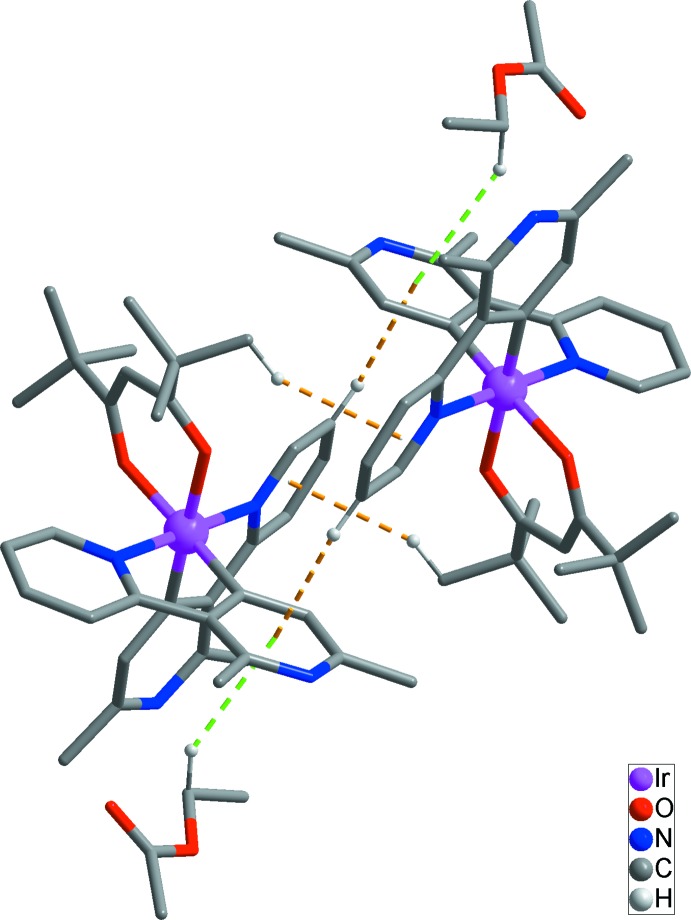
The dimeric structure of the title compound caused by C—H⋯π inter­actions between the Ir^III^ complexes (yellow dashed lines). Green dashed lines represent C—H⋯π inter­actions between ethyl acetate solvent mol­ecules and the Ir^III^ complex.

**Figure 3:**
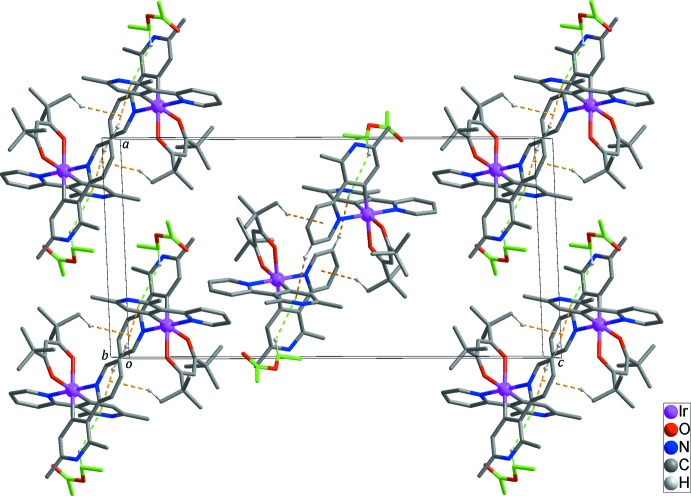
The crystal structure of the title compound: C—H⋯π inter­actions are shown as green and yellow dashed lines. The C atoms of ethyl acetate solvent mol­ecules represent are shown in green and H atoms not involved in inter­molecular inter­actions have been omitted for clarity.

In the extended structure, pairs of inversion-related Ir^III^ complexes are linked by C—H⋯π inter­actions (Table 2 ▸, yellow dashed lines in Figs. 2 ▸ and 3 ▸) between H9 with *Cg*2 and H33*A* with *Cg*1 (*Cg*1 and *Cg*2 are the centroids of the N1/C6–C10 and N4/C13–C17 rings, respectively), leading to the formation of a dimeric structure. The Ir^III^ complex mol­ecules and the ethyl acetate solvent mol­ecules are also connected by a C—H⋯π inter­actions (Table 2 ▸, green dashed lines in Fig. 2 ▸) between C38*A* and *Cg*2. No further inter­molecular inter­actions between the dimeric structures could be identified (Fig. 3 ▸).

## Thermal and luminescence properties   

**Figure 4:**
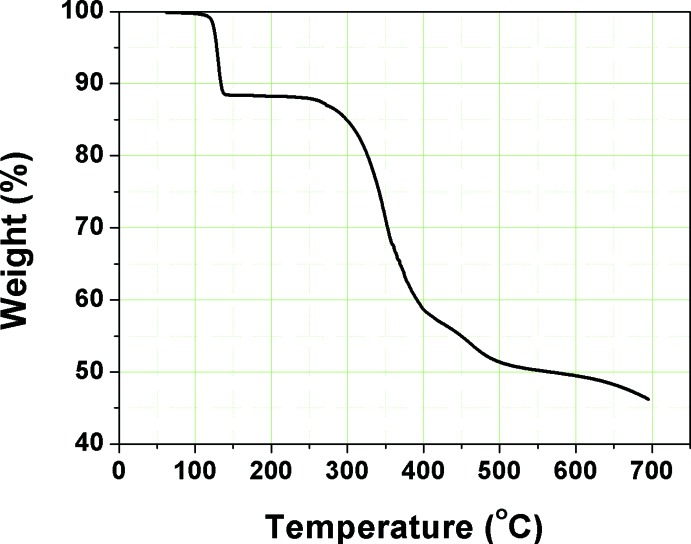
TGA curve of the title compound.

As shown in Fig. 4 ▸, the title complex has a high thermal stability. The decomposition temperature, which is defined as a 5% loss of weight, of more than 573 K is high enough to allow deposition of mol­ecules under reduced pressure without any degradation (Lee *et al.*, 2017 ▸). Thermogravimetric analysis of the title complex revealed that it was thermally stable up to 553 K. During the first stage, a significant weight loss (10%) occurred at approximately 423 K, a phenomenon that may be attributed to the loss of a subset of absorbed solvent mol­ecules as supported by crystal structure. Subsequently, a small weight loss of *ca* 5% was observed at approximately 593 K. This suggests that the complex possesses sufficient thermal stability to sublime under reduced pressure without degradation. However, it may be noted that the decomposition temperature of the title complex is lower than that of its heteroleptic analog (Lee *et al.* 2014 ▸), bis­(2′,6′-dimeth­oxy-4-methyl-2,3′-bipyridinato-*N,C* ^4^)Ir(acetyl­acetonate) (617 K). This may be due to the methyl substituents of the main bi­pyridine ligand.

**Figure 5:**
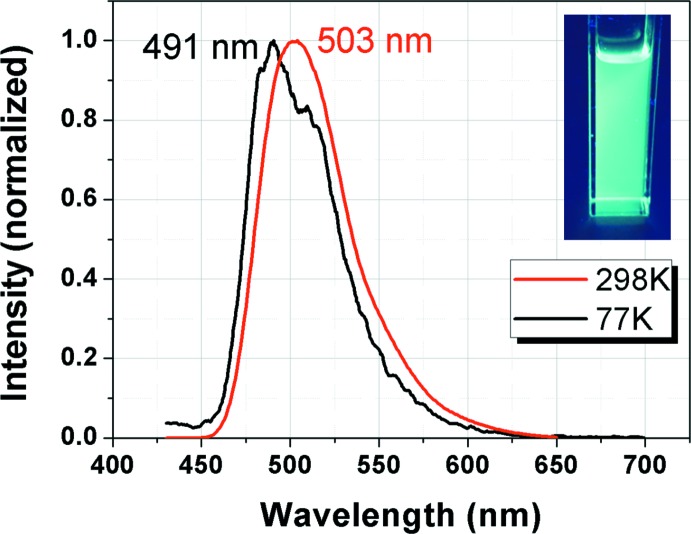
Emission spectra of the title compound at 298 K and 77 K.

The title compound displays bright bluish–green emission in solution at room temperature, as shown in Fig. 5 ▸. Emission maxima were observed at 503 nm; this wavelength is blue-shifted by approximately 10 nm from the 511 nm emission peak of mer-tris­(2′,6′-dimethyl-2,3′-bipyridinato-*κ* ^2^ *N*,*C* ^4^)iridium(III) (Kim *et al.*, 2017 ▸). Moreover, a broad and featureless emission band at 298 K was observed, indicating that this emission can be ascribed to a metal-to-ligand charge transfer (MLCT) transition (Oh *et al.*, 2014 ▸). However, a structured emission band with *λ*_max_ = 491 nm was observed at 77 K. This emission mainly originates from the ligand-centered (LC, ^3^π–π*) transition based on a previous report (Lee *et al.*, 2015 ▸). The triplet energy (*E*_T_) of the title complex was estimated to be 2.52 eV using the emission spectrum at 77 K. The quantum efficiency (Φ_PL_) of the title complex was estimated using FIrpic, bis­[2-(4,6-di­fluoro­phen­yl)pyridinato-C2,N](picolinato)iridium(III), as a standard (Φ_PL_ = 0.6) to be 0.4. The high thermal stability and good quantum efficiency of the title complex makes it a potentially useful triplet emitter for applications in OLEDs.

## Synthesis and crystallization   

All experiments were performed under a dry N_2_ atmosphere using standard Schlenk techniques. All solvents were freshly distilled over appropriate drying reagents prior to use. All starting materials were commercially purchased and used without further purification. The ^1^H NMR spectrum was recorded on a Bruker Avance 400 MHz spectrometer. The thermogravimetric spectrum was recorded on a Perkin–Elmer TGA-7 under a nitro­gen environment at a heating rate of 10 K min^−1^ over a range of 298–973 K. The Ir^III^ dimer, [(Me_2_pypy)_2_Ir(*μ*-Cl)]_2_, and the title compound were synthesized according to previous reports (Kang *et al.*, 2013 ▸). The Ir^III^ dimer, [(Me_2_pypy)_2_Ir(*μ*-Cl)]_2_, (0.15 g, 0.126 mmol), sodium carbonate (0.13 g, 1.26 mmol), and 2,2,6,6-tetra­methylhepta­ne-3,5-dione (0.066 ml, 0.32 mmol) were dissolved in THF/MeOH (1:1, 10 ml). The reaction mixture was stirred overnight at ambient temperature. All volatile components were removed under reduced pressure. The mixture was poured into EtOAc (50 ml), and then washed with water (3 × 50 ml) to remove excess sodium carbonate. Silica gel column purification with EtOAc and hexane gave a yellow powder in 60% yield. Yellow plates were recrystallized from ethyl acetate/hexane solution at low temperature. ^1^H NMR (400 MHz, CDCl_3_, δ): 8.41(*d*, *J* = 4.0 Hz, 2H), 8.08 (*d*, *J* = 4.0 Hz, 2H), 7.80 (*t*, *J* = 8.0 Hz, 2H), 7.12 (*t*, *J* = 7.9, 1 Hz, 2H), 6.02 (*s*, 2H), 5.47(*s*, 1H), 2.66 (*s*, 6H), 2.22 (*s*, 6H), 0.68 (*s*, 18H).

## Refinement   

Crystal data, data collection and structure refinement details are summarized in Table 3 ▸. All H atoms were positioned geometrically and refined using a riding model, with *d*(C—H) = 0.95 Å, *U*_iso_(H) = 1.2*U*_eq_(C) for C*sp* ^2^ H atoms, and 0.98 Å, *U*_iso_(H) = 1.5*U*_eq_(C) for methyl protons.

## Supplementary Material

Crystal structure: contains datablock(s) I, New_Global_Publ_Block. DOI: 10.1107/S2056989018011076/hb7762sup1.cif

Structure factors: contains datablock(s) I. DOI: 10.1107/S2056989018011076/hb7762Isup2.hkl

CCDC reference: 1859964

Additional supporting information:  crystallographic information; 3D view; checkCIF report

## Figures and Tables

**Table 1:** Selected bond lengths (Å)

Ir1—C1	1.974 (3)	Ir1—N1	2.032 (2)
Ir1—C13	1.977 (3)	Ir1—O1	2.132 (2)
Ir1—N3	2.028 (2)	Ir1—O2	2.136 (2)

**Table 2:** Hydrogen-bond geometry (Å, °) *Cg*1 and *Cg*2 are the centroids of the N1/C6–C10 and N4/C13–C17 rings, respectively.

*D*—H⋯*A*	*D*—H	H⋯*A*	*D*⋯*A*	*D*—H⋯*A*
C22—H22⋯O1	0.95	2.56	3.166 (4)	122
C30—H30*A*⋯O1	0.98	2.42	2.767 (5)	100
C35—H35*A*⋯O2	0.98	2.44	2.782 (6)	100
C9—H9⋯*Cg*2^i^	0.95	2.65	3.542 (4)	156
C33—H33*A*⋯*Cg*1^i^	0.98	2.76	3.624 (5)	148
C38—H38*A*⋯*Cg*2	0.98	2.85	3.711 (5)	145

Symmetry code: (i) 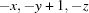.

**Table 3:** Experimental details

Crystal data	
Chemical formula	[Ir(C_11_H_19_N_2_O_2_)(C_12_H_11_N_2_)_2_]·C_4_H_8_O_2_
*M*_r_	830.02
Crystal system, space group	Monoclinic, *P*2_1_/*n*
Temperature (K)	173
*a*, *b*, *c* (Å)	13.2757 (3), 10.6258 (2), 26.3070 (5)
β (°)	92.275 (1)
*V* (Å^3^)	3708.07 (13)
*Z*	4
Radiation type	Mo *K*α
μ (mm^−1^)	3.65
Crystal size (mm)	0.36 × 0.21 × 0.05

Data collection	
Diffractometer	Bruker APEXII CCD
Absorption correction	Multi-scan (*SADABS*; Bruker, 2014 ▸)
*T*_min_, *T*_max_	0.518, 0.746
No. of measured, independent and observed [*I* > 2σ(*I*)] reflections	34241, 9158, 7635
*R*_int_	0.044
(sin θ/λ)_max_ (Å^−1^)	0.666

Refinement	
*R*[*F* ^2^ > 2σ(*F* ^2^)], *wR*(*F* ^2^), *S*	0.028, 0.065, 1.01
No. of reflections	9158
No. of parameters	435
H-atom treatment	H-atom parameters constrained
Δρ_max_, Δρ_min_ (e Å^−3^)	0.91, −0.66

Computer programs: *APEX2* and *SAINT* (Bruker, 2014 ▸), *SHELXS97* and *SHELXTL* (Sheldrick, 2008 ▸), *SHELXL2014* (Sheldrick, 2015 ▸), *DIAMOND* (Brandenburg, 2010 ▸) and *publCIF* (Westrip, 2010 ▸).

## supplementary crystallographic information

### Crystal data

**Table:**

[Ir(C_11_H_19_N_2_O_2_)(C_12_H_11_N_2_)_2_]·C_4_H_8_O_2_	*F*(000) = 1680
*M**_r_* = 830.02	*D*_x_ = 1.487 Mg m^−^^3^
Monoclinic, *P*2_1_/*n*	Mo *K*α radiation, λ = 0.71073 Å
*a* = 13.2757 (3) Å	Cell parameters from 9912 reflections
*b* = 10.6258 (2) Å	θ = 2.5–28.0°
*c* = 26.3070 (5) Å	µ = 3.65 mm^−^^1^
β = 92.275 (1)°	*T* = 173 K
*V* = 3708.07 (13) Å^3^	Plate, yellow
*Z* = 4	0.36 × 0.21 × 0.05 mm

### Data collection

**Table:**

Bruker APEXII CCD diffractometer	7635 reflections with *I* > 2σ(*I*)
φ and ω scans	*R*_int_ = 0.044
Absorption correction: multi-scan (SADABS; Bruker, 2014)	θ_max_ = 28.3°, θ_min_ = 1.6°
*T*_min_ = 0.518, *T*_max_ = 0.746	*h* = −17→17
34241 measured reflections	*k* = −14→13
9158 independent reflections	*l* = −34→35

### Refinement

**Table:**

Refinement on *F*^2^	Primary atom site location: structure-invariant direct methods
Least-squares matrix: full	Hydrogen site location: inferred from neighbouring sites
*R*[*F*^2^ > 2σ(*F*^2^)] = 0.028	H-atom parameters constrained
*wR*(*F*^2^) = 0.065	*w* = 1/[σ^2^(*F*_o_^2^) + (0.0215*P*)^2^ + 3.0807*P*] where *P* = (*F*_o_^2^ + 2*F*_c_^2^)/3
*S* = 1.01	(Δ/σ)_max_ = 0.001
9158 reflections	Δρ_max_ = 0.91 e Å^−^^3^
435 parameters	Δρ_min_ = −0.65 e Å^−^^3^
0 restraints	

### Special details

**Table:**

Geometry. All esds (except the esd in the dihedral angle between two l.s. planes) are estimated using the full covariance matrix. The cell esds are taken into account individually in the estimation of esds in distances, angles and torsion angles; correlations between esds in cell parameters are only used when they are defined by crystal symmetry. An approximate (isotropic) treatment of cell esds is used for estimating esds involving l.s. planes.

### Fractional atomic coordinates and isotropic or equivalent isotropic displacement parameters (Å^2^)

**Table:**

	*x*	*y*	*z*	*U*_iso_*/*U*_eq_	
Ir1	0.14722 (2)	0.43974 (2)	0.10988 (2)	0.01967 (4)	
O1	0.08631 (15)	0.2839 (2)	0.14947 (7)	0.0269 (5)	
O2	0.00018 (15)	0.5213 (2)	0.10965 (7)	0.0257 (5)	
N1	0.12440 (18)	0.3244 (2)	0.04888 (8)	0.0216 (5)	
N2	0.4655 (2)	0.2303 (3)	0.09802 (10)	0.0315 (6)	
N3	0.17102 (18)	0.5519 (2)	0.17158 (9)	0.0217 (5)	
N4	0.2631 (2)	0.8238 (3)	0.03719 (9)	0.0317 (6)	
C1	0.2812 (2)	0.3593 (3)	0.10869 (10)	0.0222 (6)	
C2	0.3658 (2)	0.3823 (3)	0.14065 (11)	0.0269 (7)	
H2	0.3632	0.4453	0.1662	0.032*	
C3	0.4528 (2)	0.3143 (3)	0.13543 (12)	0.0307 (7)	
C4	0.3862 (2)	0.2077 (3)	0.06524 (11)	0.0280 (7)	
C5	0.2930 (2)	0.2652 (3)	0.07071 (10)	0.0222 (6)	
C6	0.2002 (2)	0.2401 (3)	0.04003 (10)	0.0231 (6)	
C7	0.1798 (2)	0.1399 (3)	0.00727 (11)	0.0314 (7)	
H7	0.2305	0.0789	0.0017	0.038*	
C8	0.0859 (3)	0.1285 (4)	−0.01730 (14)	0.0422 (9)	
H8	0.0715	0.0586	−0.0389	0.051*	
C9	0.0135 (3)	0.2192 (4)	−0.01022 (13)	0.0408 (9)	
H9	−0.0498	0.2158	−0.0284	0.049*	
C10	0.0351 (2)	0.3143 (3)	0.02358 (11)	0.0311 (7)	
H10	−0.0152	0.3756	0.0294	0.037*	
C11	0.5423 (3)	0.3286 (4)	0.17251 (15)	0.0517 (11)	
H11A	0.5267	0.3913	0.1984	0.078*	
H11B	0.5570	0.2476	0.1890	0.078*	
H11C	0.6012	0.3562	0.1541	0.078*	
C12	0.4120 (3)	0.1192 (4)	0.02290 (13)	0.0394 (8)	
H12A	0.3846	0.1522	−0.0096	0.059*	
H12B	0.3828	0.0362	0.0292	0.059*	
H12C	0.4854	0.1117	0.0215	0.059*	
C13	0.2012 (2)	0.5906 (3)	0.07644 (10)	0.0191 (6)	
C14	0.2207 (2)	0.6071 (3)	0.02498 (10)	0.0247 (6)	
H14	0.2117	0.5386	0.0021	0.030*	
C15	0.2529 (2)	0.7220 (3)	0.00719 (11)	0.0277 (7)	
C16	0.2461 (2)	0.8110 (3)	0.08704 (11)	0.0296 (7)	
C17	0.2183 (2)	0.6962 (3)	0.10817 (10)	0.0229 (6)	
C18	0.2029 (2)	0.6714 (3)	0.16275 (10)	0.0240 (6)	
C19	0.2196 (3)	0.7524 (4)	0.20390 (12)	0.0418 (9)	
H19	0.2456	0.8344	0.1986	0.050*	
C20	0.1984 (3)	0.7133 (4)	0.25240 (12)	0.0495 (11)	
H20	0.2105	0.7683	0.2805	0.059*	
C21	0.1599 (3)	0.5955 (4)	0.26008 (11)	0.0373 (8)	
H21	0.1412	0.5693	0.2929	0.045*	
C22	0.1493 (2)	0.5164 (3)	0.21898 (11)	0.0293 (7)	
H22	0.1256	0.4332	0.2242	0.035*	
C23	0.2756 (3)	0.7427 (4)	−0.04770 (12)	0.0415 (9)	
H23A	0.2662	0.6637	−0.0665	0.062*	
H23B	0.2299	0.8068	−0.0623	0.062*	
H23C	0.3455	0.7712	−0.0501	0.062*	
C24	0.2582 (5)	0.9326 (4)	0.11632 (15)	0.0646 (15)	
H24A	0.2001	0.9446	0.1377	0.097*	
H24B	0.2622	1.0030	0.0924	0.097*	
H24C	0.3200	0.9292	0.1379	0.097*	
C25	−0.0311 (3)	0.1427 (3)	0.18262 (13)	0.0373 (8)	
C26	−0.0038 (2)	0.2754 (3)	0.16426 (11)	0.0274 (7)	
C27	−0.0740 (2)	0.3723 (3)	0.16390 (12)	0.0317 (7)	
H27	−0.1331	0.3575	0.1822	0.038*	
C28	−0.0679 (2)	0.4890 (3)	0.13971 (11)	0.0253 (6)	
C29	−0.1472 (2)	0.5922 (3)	0.14756 (12)	0.0319 (7)	
C30	0.0530 (4)	0.0920 (4)	0.21790 (16)	0.0561 (12)	
H30A	0.1174	0.0982	0.2011	0.084*	
H30B	0.0563	0.1414	0.2494	0.084*	
H30C	0.0394	0.0037	0.2259	0.084*	
C31	−0.0357 (4)	0.0615 (4)	0.13371 (17)	0.0585 (12)	
H31A	0.0292	0.0662	0.1172	0.088*	
H31B	−0.0499	−0.0261	0.1426	0.088*	
H31C	−0.0892	0.0932	0.1104	0.088*	
C32	−0.1313 (4)	0.1344 (4)	0.2074 (2)	0.0702 (15)	
H32A	−0.1843	0.1674	0.1841	0.105*	
H32B	−0.1458	0.0463	0.2153	0.105*	
H32C	−0.1289	0.1840	0.2388	0.105*	
C33	−0.1998 (4)	0.6203 (5)	0.09674 (16)	0.0679 (15)	
H33A	−0.1497	0.6438	0.0721	0.102*	
H33B	−0.2365	0.5454	0.0846	0.102*	
H33C	−0.2473	0.6899	0.1006	0.102*	
C34	−0.2262 (4)	0.5567 (5)	0.1841 (2)	0.089 (2)	
H34A	−0.2627	0.4822	0.1714	0.134*	
H34B	−0.1940	0.5379	0.2174	0.134*	
H34C	−0.2736	0.6267	0.1874	0.134*	
C35	−0.0905 (4)	0.7068 (5)	0.1668 (2)	0.091 (2)	
H35A	−0.0391	0.7295	0.1427	0.136*	
H35B	−0.1376	0.7771	0.1700	0.136*	
H35C	−0.0581	0.6883	0.2001	0.136*	
O3	0.4888 (5)	0.8350 (6)	0.17875 (19)	0.143 (2)	
O4	0.5575 (2)	0.7781 (3)	0.10749 (13)	0.0626 (8)	
C36	0.6151 (5)	0.9681 (6)	0.1430 (3)	0.095 (2)	
H36A	0.6557	0.9607	0.1128	0.143*	
H36B	0.5739	1.0445	0.1404	0.143*	
H36C	0.6598	0.9728	0.1735	0.143*	
C37	0.5479 (5)	0.8554 (6)	0.1465 (2)	0.0797 (16)	
C38	0.4976 (4)	0.6650 (5)	0.1056 (2)	0.0765 (15)	
H38A	0.4256	0.6868	0.1090	0.092*	
H38B	0.5184	0.6093	0.1343	0.092*	
C39	0.5104 (4)	0.5992 (6)	0.0575 (2)	0.0815 (17)	
H39A	0.4693	0.5226	0.0566	0.122*	
H39B	0.4890	0.6542	0.0291	0.122*	
H39C	0.5815	0.5768	0.0544	0.122*	

### Atomic displacement parameters (Å^2^)

**Table:**

	*U*^11^	*U*^22^	*U*^33^	*U*^12^	*U*^13^	*U*^23^
Ir1	0.02112 (6)	0.01750 (7)	0.02056 (6)	0.00037 (5)	0.00316 (4)	−0.00117 (5)
O1	0.0271 (11)	0.0224 (12)	0.0316 (10)	−0.0005 (9)	0.0066 (8)	0.0018 (9)
O2	0.0246 (11)	0.0238 (12)	0.0291 (10)	0.0032 (9)	0.0053 (8)	0.0019 (9)
N1	0.0232 (12)	0.0193 (13)	0.0224 (11)	−0.0016 (10)	0.0021 (9)	−0.0022 (10)
N2	0.0262 (14)	0.0265 (16)	0.0417 (14)	0.0043 (12)	0.0020 (11)	−0.0018 (13)
N3	0.0237 (12)	0.0198 (14)	0.0217 (11)	0.0040 (10)	0.0016 (9)	−0.0010 (10)
N4	0.0421 (16)	0.0253 (15)	0.0281 (12)	−0.0068 (13)	0.0046 (11)	0.0038 (12)
C1	0.0265 (15)	0.0152 (15)	0.0247 (13)	0.0009 (12)	−0.0007 (11)	0.0012 (12)
C2	0.0284 (16)	0.0256 (18)	0.0266 (14)	−0.0002 (14)	0.0003 (12)	−0.0032 (13)
C3	0.0271 (16)	0.0301 (19)	0.0346 (15)	0.0034 (14)	−0.0031 (12)	0.0005 (15)
C4	0.0312 (16)	0.0184 (17)	0.0347 (15)	0.0026 (13)	0.0053 (13)	−0.0014 (13)
C5	0.0262 (15)	0.0182 (16)	0.0224 (13)	0.0015 (12)	0.0031 (11)	0.0014 (12)
C6	0.0258 (15)	0.0190 (16)	0.0248 (13)	−0.0015 (12)	0.0047 (11)	−0.0004 (12)
C7	0.0339 (18)	0.0269 (19)	0.0333 (15)	0.0018 (14)	0.0015 (13)	−0.0105 (14)
C8	0.044 (2)	0.036 (2)	0.0453 (19)	−0.0050 (17)	−0.0053 (16)	−0.0207 (18)
C9	0.0332 (19)	0.044 (2)	0.0442 (18)	−0.0011 (17)	−0.0085 (15)	−0.0131 (17)
C10	0.0257 (16)	0.032 (2)	0.0349 (15)	0.0018 (14)	−0.0016 (12)	−0.0068 (15)
C11	0.035 (2)	0.057 (3)	0.062 (2)	0.0096 (19)	−0.0171 (18)	−0.016 (2)
C12	0.0342 (19)	0.037 (2)	0.0470 (19)	0.0074 (16)	0.0076 (15)	−0.0154 (17)
C13	0.0138 (13)	0.0213 (16)	0.0221 (12)	0.0026 (11)	0.0010 (10)	0.0009 (11)
C14	0.0279 (16)	0.0258 (17)	0.0205 (13)	−0.0020 (13)	0.0016 (11)	−0.0035 (12)
C15	0.0277 (16)	0.0307 (19)	0.0247 (13)	−0.0026 (14)	0.0017 (12)	0.0026 (13)
C16	0.0407 (18)	0.0207 (17)	0.0275 (14)	−0.0033 (15)	0.0013 (13)	−0.0011 (14)
C17	0.0235 (14)	0.0222 (17)	0.0229 (12)	0.0012 (12)	0.0012 (11)	−0.0013 (12)
C18	0.0241 (15)	0.0238 (17)	0.0242 (13)	0.0002 (13)	0.0017 (11)	−0.0019 (13)
C19	0.068 (3)	0.028 (2)	0.0289 (16)	−0.0140 (19)	0.0041 (16)	−0.0051 (15)
C20	0.086 (3)	0.039 (2)	0.0234 (15)	−0.008 (2)	0.0026 (17)	−0.0125 (16)
C21	0.053 (2)	0.038 (2)	0.0205 (14)	0.0046 (17)	0.0035 (14)	0.0006 (14)
C22	0.0349 (18)	0.0289 (18)	0.0242 (14)	0.0035 (14)	0.0005 (12)	0.0041 (13)
C23	0.054 (2)	0.043 (2)	0.0272 (15)	−0.0132 (19)	0.0055 (15)	0.0030 (16)
C24	0.136 (5)	0.024 (2)	0.0346 (19)	−0.018 (3)	0.012 (2)	−0.0032 (17)
C25	0.047 (2)	0.027 (2)	0.0385 (17)	−0.0080 (16)	0.0078 (15)	0.0058 (15)
C26	0.0340 (17)	0.0257 (18)	0.0228 (13)	−0.0059 (14)	0.0034 (12)	−0.0001 (13)
C27	0.0283 (17)	0.032 (2)	0.0358 (16)	−0.0040 (15)	0.0125 (13)	0.0015 (15)
C28	0.0249 (15)	0.0259 (17)	0.0254 (14)	0.0006 (13)	0.0032 (11)	−0.0071 (13)
C29	0.0290 (17)	0.032 (2)	0.0356 (16)	0.0057 (14)	0.0086 (13)	−0.0058 (15)
C30	0.079 (3)	0.041 (3)	0.049 (2)	−0.006 (2)	0.003 (2)	0.018 (2)
C31	0.083 (3)	0.034 (2)	0.058 (2)	−0.016 (2)	0.005 (2)	−0.007 (2)
C32	0.070 (3)	0.042 (3)	0.102 (4)	−0.015 (2)	0.043 (3)	0.014 (3)
C33	0.063 (3)	0.090 (4)	0.051 (2)	0.047 (3)	0.005 (2)	0.003 (3)
C34	0.082 (4)	0.071 (4)	0.120 (5)	0.037 (3)	0.078 (4)	0.033 (3)
C35	0.059 (3)	0.051 (3)	0.161 (6)	0.016 (3)	−0.012 (3)	−0.064 (4)
O3	0.181 (6)	0.148 (5)	0.103 (3)	−0.029 (4)	0.045 (4)	−0.011 (4)
O4	0.0454 (17)	0.054 (2)	0.089 (2)	0.0014 (15)	0.0076 (16)	0.0046 (18)
C36	0.097 (5)	0.067 (4)	0.121 (5)	0.008 (4)	−0.014 (4)	−0.008 (4)
C37	0.075 (4)	0.082 (5)	0.081 (4)	0.007 (3)	0.001 (3)	0.016 (3)
C38	0.050 (3)	0.067 (4)	0.113 (4)	−0.007 (3)	0.005 (3)	0.019 (3)
C39	0.059 (3)	0.074 (4)	0.110 (5)	−0.017 (3)	−0.013 (3)	0.009 (4)

### Geometric parameters (Å, º)

**Table:**

Ir1—C1	1.974 (3)	C21—C22	1.373 (5)
Ir1—C13	1.977 (3)	C21—H21	0.9500
Ir1—N3	2.028 (2)	C22—H22	0.9500
Ir1—N1	2.032 (2)	C23—H23A	0.9800
Ir1—O1	2.132 (2)	C23—H23B	0.9800
Ir1—O2	2.136 (2)	C23—H23C	0.9800
O1—C26	1.276 (4)	C24—H24A	0.9800
O2—C28	1.271 (3)	C24—H24B	0.9800
N1—C10	1.340 (4)	C24—H24C	0.9800
N1—C6	1.374 (4)	C25—C32	1.507 (5)
N2—C3	1.344 (4)	C25—C30	1.521 (6)
N2—C4	1.355 (4)	C25—C26	1.539 (5)
N3—C22	1.345 (4)	C25—C31	1.548 (5)
N3—C18	1.362 (4)	C26—C27	1.388 (5)
N4—C15	1.343 (4)	C27—C28	1.398 (5)
N4—C16	1.346 (4)	C27—H27	0.9500
C1—C2	1.398 (4)	C28—C29	1.540 (4)
C1—C5	1.427 (4)	C29—C34	1.499 (5)
C2—C3	1.373 (4)	C29—C35	1.508 (6)
C2—H2	0.9500	C29—C33	1.513 (5)
C3—C11	1.515 (4)	C30—H30A	0.9800
C4—C5	1.393 (4)	C30—H30B	0.9800
C4—C12	1.508 (4)	C30—H30C	0.9800
C5—C6	1.471 (4)	C31—H31A	0.9800
C6—C7	1.389 (4)	C31—H31B	0.9800
C7—C8	1.386 (5)	C31—H31C	0.9800
C7—H7	0.9500	C32—H32A	0.9800
C8—C9	1.379 (5)	C32—H32B	0.9800
C8—H8	0.9500	C32—H32C	0.9800
C9—C10	1.368 (5)	C33—H33A	0.9800
C9—H9	0.9500	C33—H33B	0.9800
C10—H10	0.9500	C33—H33C	0.9800
C11—H11A	0.9800	C34—H34A	0.9800
C11—H11B	0.9800	C34—H34B	0.9800
C11—H11C	0.9800	C34—H34C	0.9800
C12—H12A	0.9800	C35—H35A	0.9800
C12—H12B	0.9800	C35—H35B	0.9800
C12—H12C	0.9800	C35—H35C	0.9800
C13—C14	1.399 (4)	O3—C37	1.199 (7)
C13—C17	1.412 (4)	O4—C37	1.324 (7)
C14—C15	1.381 (4)	O4—C38	1.441 (6)
C14—H14	0.9500	C36—C37	1.498 (8)
C15—C23	1.503 (4)	C36—H36A	0.9800
C16—C17	1.396 (4)	C36—H36B	0.9800
C16—C24	1.509 (5)	C36—H36C	0.9800
C17—C18	1.482 (4)	C38—C39	1.462 (7)
C18—C19	1.394 (4)	C38—H38A	0.9900
C19—C20	1.381 (5)	C38—H38B	0.9900
C19—H19	0.9500	C39—H39A	0.9800
C20—C21	1.370 (5)	C39—H39B	0.9800
C20—H20	0.9500	C39—H39C	0.9800
			
C1—Ir1—C13	90.08 (12)	N3—C22—C21	122.6 (3)
C1—Ir1—N3	98.92 (11)	N3—C22—H22	118.7
C13—Ir1—N3	80.33 (11)	C21—C22—H22	118.7
C1—Ir1—N1	80.37 (11)	C15—C23—H23A	109.5
C13—Ir1—N1	100.53 (10)	C15—C23—H23B	109.5
N3—Ir1—N1	178.87 (10)	H23A—C23—H23B	109.5
C1—Ir1—O1	91.77 (10)	C15—C23—H23C	109.5
C13—Ir1—O1	176.65 (10)	H23A—C23—H23C	109.5
N3—Ir1—O1	96.62 (9)	H23B—C23—H23C	109.5
N1—Ir1—O1	82.53 (9)	C16—C24—H24A	109.5
C1—Ir1—O2	178.00 (10)	C16—C24—H24B	109.5
C13—Ir1—O2	90.96 (10)	H24A—C24—H24B	109.5
N3—Ir1—O2	82.94 (9)	C16—C24—H24C	109.5
N1—Ir1—O2	97.76 (9)	H24A—C24—H24C	109.5
O1—Ir1—O2	87.27 (8)	H24B—C24—H24C	109.5
C26—O1—Ir1	125.6 (2)	C32—C25—C30	110.8 (3)
C28—O2—Ir1	124.1 (2)	C32—C25—C26	114.3 (3)
C10—N1—C6	120.1 (3)	C30—C25—C26	109.9 (3)
C10—N1—Ir1	122.8 (2)	C32—C25—C31	108.7 (4)
C6—N1—Ir1	116.10 (18)	C30—C25—C31	108.3 (3)
C3—N2—C4	117.8 (3)	C26—C25—C31	104.6 (3)
C22—N3—C18	119.9 (3)	O1—C26—C27	125.7 (3)
C22—N3—Ir1	123.0 (2)	O1—C26—C25	113.3 (3)
C18—N3—Ir1	116.82 (18)	C27—C26—C25	121.0 (3)
C15—N4—C16	118.3 (3)	C26—C27—C28	127.5 (3)
C2—C1—C5	115.8 (3)	C26—C27—H27	116.2
C2—C1—Ir1	128.2 (2)	C28—C27—H27	116.2
C5—C1—Ir1	116.0 (2)	O2—C28—C27	125.4 (3)
C3—C2—C1	120.5 (3)	O2—C28—C29	113.4 (3)
C3—C2—H2	119.8	C27—C28—C29	121.2 (3)
C1—C2—H2	119.8	C34—C29—C35	109.9 (4)
N2—C3—C2	123.5 (3)	C34—C29—C33	107.8 (4)
N2—C3—C11	114.8 (3)	C35—C29—C33	110.1 (4)
C2—C3—C11	121.6 (3)	C34—C29—C28	114.0 (3)
N2—C4—C5	121.8 (3)	C35—C29—C28	106.6 (3)
N2—C4—C12	112.8 (3)	C33—C29—C28	108.4 (3)
C5—C4—C12	125.4 (3)	C25—C30—H30A	109.5
C4—C5—C1	120.2 (3)	C25—C30—H30B	109.5
C4—C5—C6	126.3 (3)	H30A—C30—H30B	109.5
C1—C5—C6	113.5 (3)	C25—C30—H30C	109.5
N1—C6—C7	118.6 (3)	H30A—C30—H30C	109.5
N1—C6—C5	113.1 (3)	H30B—C30—H30C	109.5
C7—C6—C5	128.2 (3)	C25—C31—H31A	109.5
C8—C7—C6	120.4 (3)	C25—C31—H31B	109.5
C8—C7—H7	119.8	H31A—C31—H31B	109.5
C6—C7—H7	119.8	C25—C31—H31C	109.5
C9—C8—C7	119.6 (3)	H31A—C31—H31C	109.5
C9—C8—H8	120.2	H31B—C31—H31C	109.5
C7—C8—H8	120.2	C25—C32—H32A	109.5
C10—C9—C8	118.3 (3)	C25—C32—H32B	109.5
C10—C9—H9	120.8	H32A—C32—H32B	109.5
C8—C9—H9	120.8	C25—C32—H32C	109.5
N1—C10—C9	122.7 (3)	H32A—C32—H32C	109.5
N1—C10—H10	118.6	H32B—C32—H32C	109.5
C9—C10—H10	118.6	C29—C33—H33A	109.5
C3—C11—H11A	109.5	C29—C33—H33B	109.5
C3—C11—H11B	109.5	H33A—C33—H33B	109.5
H11A—C11—H11B	109.5	C29—C33—H33C	109.5
C3—C11—H11C	109.5	H33A—C33—H33C	109.5
H11A—C11—H11C	109.5	H33B—C33—H33C	109.5
H11B—C11—H11C	109.5	C29—C34—H34A	109.5
C4—C12—H12A	109.5	C29—C34—H34B	109.5
C4—C12—H12B	109.5	H34A—C34—H34B	109.5
H12A—C12—H12B	109.5	C29—C34—H34C	109.5
C4—C12—H12C	109.5	H34A—C34—H34C	109.5
H12A—C12—H12C	109.5	H34B—C34—H34C	109.5
H12B—C12—H12C	109.5	C29—C35—H35A	109.5
C14—C13—C17	116.2 (3)	C29—C35—H35B	109.5
C14—C13—Ir1	128.1 (2)	H35A—C35—H35B	109.5
C17—C13—Ir1	115.74 (19)	C29—C35—H35C	109.5
C15—C14—C13	120.7 (3)	H35A—C35—H35C	109.5
C15—C14—H14	119.7	H35B—C35—H35C	109.5
C13—C14—H14	119.7	C37—O4—C38	118.3 (4)
N4—C15—C14	122.6 (3)	C37—C36—H36A	109.5
N4—C15—C23	115.3 (3)	C37—C36—H36B	109.5
C14—C15—C23	122.1 (3)	H36A—C36—H36B	109.5
N4—C16—C17	122.2 (3)	C37—C36—H36C	109.5
N4—C16—C24	113.1 (3)	H36A—C36—H36C	109.5
C17—C16—C24	124.7 (3)	H36B—C36—H36C	109.5
C16—C17—C13	119.9 (2)	O3—C37—O4	121.5 (6)
C16—C17—C18	126.2 (3)	O3—C37—C36	126.6 (7)
C13—C17—C18	114.0 (3)	O4—C37—C36	111.9 (5)
N3—C18—C19	119.0 (3)	O4—C38—C39	110.2 (4)
N3—C18—C17	112.8 (3)	O4—C38—H38A	109.6
C19—C18—C17	128.2 (3)	C39—C38—H38A	109.6
C20—C19—C18	120.0 (3)	O4—C38—H38B	109.6
C20—C19—H19	120.0	C39—C38—H38B	109.6
C18—C19—H19	120.0	H38A—C38—H38B	108.1
C21—C20—C19	120.1 (3)	C38—C39—H39A	109.5
C21—C20—H20	119.9	C38—C39—H39B	109.5
C19—C20—H20	119.9	H39A—C39—H39B	109.5
C20—C21—C22	118.1 (3)	C38—C39—H39C	109.5
C20—C21—H21	120.9	H39A—C39—H39C	109.5
C22—C21—H21	120.9	H39B—C39—H39C	109.5
			
C5—C1—C2—C3	−1.5 (5)	C14—C13—C17—C16	4.4 (4)
Ir1—C1—C2—C3	177.9 (2)	Ir1—C13—C17—C16	−173.2 (2)
C4—N2—C3—C2	−3.2 (5)	C14—C13—C17—C18	−176.2 (3)
C4—N2—C3—C11	176.3 (3)	Ir1—C13—C17—C18	6.2 (3)
C1—C2—C3—N2	5.2 (5)	C22—N3—C18—C19	4.7 (4)
C1—C2—C3—C11	−174.3 (3)	Ir1—N3—C18—C19	179.5 (2)
C3—N2—C4—C5	−2.4 (5)	C22—N3—C18—C17	−177.0 (3)
C3—N2—C4—C12	175.8 (3)	Ir1—N3—C18—C17	−2.2 (3)
N2—C4—C5—C1	5.8 (5)	C16—C17—C18—N3	176.8 (3)
C12—C4—C5—C1	−172.1 (3)	C13—C17—C18—N3	−2.5 (4)
N2—C4—C5—C6	−174.8 (3)	C16—C17—C18—C19	−5.1 (5)
C12—C4—C5—C6	7.3 (5)	C13—C17—C18—C19	175.6 (3)
C2—C1—C5—C4	−3.7 (4)	N3—C18—C19—C20	−3.7 (5)
Ir1—C1—C5—C4	176.8 (2)	C17—C18—C19—C20	178.3 (4)
C2—C1—C5—C6	176.8 (3)	C18—C19—C20—C21	−0.7 (6)
Ir1—C1—C5—C6	−2.7 (3)	C19—C20—C21—C22	3.8 (6)
C10—N1—C6—C7	−4.5 (4)	C18—N3—C22—C21	−1.5 (5)
Ir1—N1—C6—C7	164.7 (2)	Ir1—N3—C22—C21	−176.0 (3)
C10—N1—C6—C5	179.7 (3)	C20—C21—C22—N3	−2.9 (5)
Ir1—N1—C6—C5	−11.2 (3)	Ir1—O1—C26—C27	−9.9 (4)
C4—C5—C6—N1	−170.5 (3)	Ir1—O1—C26—C25	169.11 (19)
C1—C5—C6—N1	8.9 (4)	C32—C25—C26—O1	171.5 (3)
C4—C5—C6—C7	14.1 (5)	C30—C25—C26—O1	46.2 (4)
C1—C5—C6—C7	−166.4 (3)	C31—C25—C26—O1	−69.8 (4)
N1—C6—C7—C8	2.3 (5)	C32—C25—C26—C27	−9.5 (5)
C5—C6—C7—C8	177.5 (3)	C30—C25—C26—C27	−134.7 (3)
C6—C7—C8—C9	1.9 (6)	C31—C25—C26—C27	109.2 (4)
C7—C8—C9—C10	−4.0 (6)	O1—C26—C27—C28	12.3 (5)
C6—N1—C10—C9	2.4 (5)	C25—C26—C27—C28	−166.6 (3)
Ir1—N1—C10—C9	−166.0 (3)	Ir1—O2—C28—C27	−24.2 (4)
C8—C9—C10—N1	1.9 (6)	Ir1—O2—C28—C29	156.3 (2)
C17—C13—C14—C15	−1.2 (4)	C26—C27—C28—O2	7.1 (6)
Ir1—C13—C14—C15	176.0 (2)	C26—C27—C28—C29	−173.5 (3)
C16—N4—C15—C14	3.5 (5)	O2—C28—C29—C34	−178.3 (4)
C16—N4—C15—C23	−178.5 (3)	C27—C28—C29—C34	2.2 (5)
C13—C14—C15—N4	−2.8 (5)	O2—C28—C29—C35	−56.9 (4)
C13—C14—C15—C23	179.3 (3)	C27—C28—C29—C35	123.6 (4)
C15—N4—C16—C17	−0.2 (5)	O2—C28—C29—C33	61.6 (4)
C15—N4—C16—C24	−179.0 (3)	C27—C28—C29—C33	−117.9 (4)
N4—C16—C17—C13	−3.9 (5)	C38—O4—C37—O3	−2.3 (8)
C24—C16—C17—C13	174.8 (4)	C38—O4—C37—C36	179.9 (4)
N4—C16—C17—C18	176.8 (3)	C37—O4—C38—C39	173.9 (4)
C24—C16—C17—C18	−4.5 (6)		

### Hydrogen-bond geometry (Å, º)

*Cg*1 and *Cg*2 are the centroids of the N1/C6–C10 and N4/C13–C17 rings, respectively.

**Table:**

*D*—H···*A*	*D*—H	H···*A*	*D*···*A*	*D*—H···*A*
C22—H22···O1	0.95	2.56	3.166 (4)	122
C30—H30*A*···O1	0.98	2.42	2.767 (5)	100
C35—H35*A*···O2	0.98	2.44	2.782 (6)	100
C9—H9···*Cg*2^i^	0.95	2.65	3.542 (4)	156
C33—H33*A*···*Cg*1^i^	0.98	2.76	3.624 (5)	148
C38—H38*A*···*Cg*2	0.98	2.85	3.711 (5)	145

Symmetry code: (i) −*x*, −*y*+1, −*z*.
